# Ultrasonic Transducers for In-Service Inspection and Continuous Monitoring in High-Temperature Environments

**DOI:** 10.3390/s23073520

**Published:** 2023-03-28

**Authors:** Sevan Bouchy, Ricardo J. Zednik, Pierre Belanger

**Affiliations:** 1 PULETS, École de Technologie Supérieure (ÉTS), Montréal, QC H3C 1K3, Canada; 2 Department of Mechanical Engineering, École de Technologie Supérieure (ÉTS), Montréal, QC H3C 1K3, Canada

**Keywords:** bulk acoustic wave sensors, continuous inspection, nondestructive evaluation, high temperature, in-service inspection, piezoelectric, transducer, ultrasonic sensors, ultrasound

## Abstract

The inspection of structures operating at high temperatures is a major challenge in a variety of industries, including the energy and petrochemical industries. Operators are typically performing nondestructive evaluations using ultrasound to monitor component thicknesses during scheduled shutdowns, thereby ensuring safe operation of their plants. However, despite being costly, this calendar-based approach may lead to undetected corrosion, which can potentially result in catastrophic failures. There is therefore a need for ultrasonic transducers designed to withstand permanent exposure to high temperatures, so as to continuously monitor the remnant thicknesses of structures in real time. This paper discusses the design of a heat-resistant ultrasonic transducer based on a piezoelectric element. The piezoelectric material, the electrodes, the backing layer, the wires and the casing are presented in detail from the acoustic and thermal expansion point of view. Four transducers optimized for 3 MHz were manufactured and tested to destruction in different conditions: (1) 72-h temperature steps from room temperature to 750 ${}^{\circ }$C, (2) thermal cycles from room temperature to 500 ${}^{\circ }$C and (3) 60 days of continuous operation at >550 ${}^{\circ }$C. The paper discusses the results, as well as the effect of temperature over time on the properties of the transducer.

## 1. Introduction

Industry often operates high-temperature processes (≥500 ${}^{\circ }$C) that include corrosive products, particularly in the oil, gas and petrochemical fields. In some instances, this is further complicated by elevated processing pressures (≥150 bar). Elevated temperatures and pressures can then lead to accelerated erosion and corrosion, leading to the rapid thinning of components including pipework. In such conditions, regular calendar-based thickness gauging campaigns are typically employed. However, calendar-based inspections cannot track the progress of corrosion between measurement campaigns, and catastrophic failures of pipework (with considerable economic, health and environmental costs) regularly occur in the oil and gas industry: approximately three such catastrophic accidents occur per year within the European Union (EU) and the Organization for Economic Cooperation and Development (OECD) countries [1].

Current technology requires costly shutdowns of plants during thickness gauging campaigns to ensure that the inspectors can safely perform the measurements and the ultrasonic testing transducers can operate. Point-by-point ultrasonic thickness gauging remains the gold standard to determine the remnant thickness of pipework. Conventional piezoelectric or electromagnetic acoustic transducers (EMAT) are typically used in ultrasonic thickness gauging. However, the Maximum Operating Temperature (MOT) of both types of transducers is limited to ≈200–300 ${}^{\circ }$C because of the piezoelectric element or the magnet [2]. Over the last decade, much effort has been expended to develop novel transducers designed to withstand harsh environments for permanent installation scenarios that can be left in situ and therefore avoid plant shutdowns. For example, Cegla et al. [3] suggested the use of a waveguide to place the temperature-sensitive elements of the piezoelectric transducer away from the heat source. However, the waveguide approach cannot be used when the space around the pipework is restricted or when the temperature remains high at a distance of approximately 50 cm away from the structure to be inspected. As an alternative, a water or air cooling system, such as the one proposed by Burrows et al. [4], can be used. The addition of a cooling system, however, can lead to difficulty in the interpretation of the received signals due to the temperature gradient between the transducer and the surface inspected, and also it adds a new point of failure to the transducer. Other authors [5] proposed a piezoelectric transducer concept able to operate at temperatures as high as 800 ${}^{\circ }$C without cooling and presenting a small footprint, but were unable to demonstrate operation beyond 72 h of use. More recently, a high-temperature EMAT was designed to withstand a permanent installation up to 450 ${}^{\circ }$C without cooling [6]. The concept was shown to have stable performance after one month of continuous exposure at 450 ${}^{\circ }$C. However, the experiments were only conducted on aluminum and magnetite-coated steel samples. Therefore, the development of a small ultrasonic transducer designed to be permanently installed and able to survive multiple thermal cycles, as well as long-term exposure to temperatures above 500 ${}^{\circ }$C without ancillary cooling, remains an unsolved challenge.

This paper presents a compact piezoelectric ultrasonic transducer designed to be permanently exposed to high temperatures. The selection of the different components is discussed in detail in terms of the acoustic and thermal expansion properties. Four transducers optimized for 3 MHz (near a typical ultrasound frequency) were manufactured and tested to destruction in three different scenarios that simulated various operational environments: (1) 72-h increasing temperature from 50 ${}^{\circ }$C to 750 ${}^{\circ }$C, (2) thermal cycles between room temperature and 500 ${}^{\circ }$C and (3) continuous exposure to 550 ${}^{\circ }$C for 60 days.

## 2. Transducer Design

**Figure 1:**
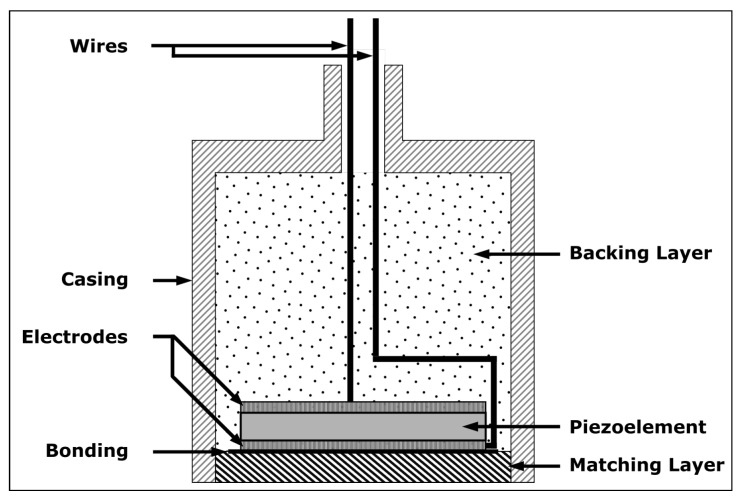
Schematic cross-sectional view of an ultrasonic transducer showing its components.

The schematic of an ultrasonic piezoelectric transducer is illustrated in Figure 1, showing the piezoelectric material, the electrodes, the backing layer, the matching layer, the wires and the outer casing/packaging.

The decision regarding the materials depends on the acoustic impedance, acoustic coupling between each layer, absorbing or transmitting characteristics, thermal stability and coefficients of thermal expansion. The selection of the different layers must therefore be coordinated with the application. In the present case, material layers were selected to resemble traditional ultrasound devices as much as possible given the temperature exposure.

### 2.1. The Piezoelectric Element

The piezoelectric element is the key component of the piezoelectric transducer. Due to the piezoelectric effect, it converts the applied electrical voltage to mechanical displacement (transmitter) or mechanical displacement to the voltage (receiver). All other transducer layers are selected according to this material and the material to be inspected. The choice of the piezoelectric material defines, in part, the performance of the resulting transducer. The geometry and orientation of the material determine the resonant frequency and the type of wave to be generated (longitudinal, transversal, shear or torsional). Generally, the emission of a single type of wave at a specific frequency simplifies the analysis of the resulting signal. Nondestructive evaluation (NDE) often requires short signals or low ringing (in the time domain) containing information over a wide frequency band to obtain a good temporal resolution. Single-crystal piezoelectric elements generally exhibit low internal damping (high value of mechanical quality factor Q), such that a relatively long ringdown response is seen for a pulse voltage excitation. This can be solved by designing a proper backing. Lead zirconium titanate (PZT) 5A material is one of the most common materials. PZT-5A has piezoelectric coefficients of $d_{15}=460$ pC/N, an electromechanical coupling factor of $k_{15}=0.61$, a Curie temperature of 350 ${}^{\circ }$C [7] and a Figure of Merit (FoM) of 14.6 [8]—this is a dimensionless index that can be used to compare piezoelectric materials in pulse-echo mode.

At elevated temperatures, lithium niobate (LiNbO${}_{3}$) is considered one of the most promising candidates for realizing a piezoelectric transducer [9,10] because it has large piezoelectric coefficients of $d_{22^{\prime }}=38.5$ pC/N, a large electromechanical coupling factor of $k_{22^{\prime }}=0.485$, a high Curie temperature of 1210 ${}^{\circ }$C [11,12] and an FoM of 1.5 [8]. However, an efficient backing layer is required to attenuate the long resonance in the time domain due to its high quality factor. The experimental observation of piezoelectricity in LiNbO${}_{3}$ was found to often degrade well below the Curie temperature due to its oxygen loss or chemical deterioration [2,13,14,15]. These observations were recently confirmed to be caused by internal shorting of the crystal due to electrical conductivity at elevated temperatures dominated by Li+ ion motion and the composition of the LiNbO${}_{3}$ element. However, it was shown that it may be overcome by operating at a high frequency (>MHz) and choosing a monocrystal or a stoichiometric element [16]. The complete set of elastic, piezoelectric and dielectric coefficients has been evaluated from room temperature to 900 ${}^{\circ }$C [17,18]. The selected material, 36${}^{\circ }$ Y-cut LiNbO${}_{3}$ (from MTI Corporation), was chosen because its thickness vibration mode is quasi-extensional, with the geometry selected to ensure maximum resonance at the desired mode and minimal at other modes. Indeed, the k${}_{22^{\prime }}$ of the quasi-shear wave is close to 0 [19]. The selected material has the following parameters, which are used to match the materials for each layer to build a high-temperature ultrasonic transducer: its coefficients of thermal expansion (CTE) are $\alpha_{11}$ = 14.86 × 10${}^{-6}$/${}^{\circ }$C and $\alpha_{33}$ = 6.54 × 10${}^{-6}$/${}^{\circ }$C, its speed of sound v = 7340 m/s, its density is ρ = 4.65 g/cm${}^{3}$ and so its acoustic impedance Z = 34 MRayl [20]. Indeed, the acoustic impedance formula is expressed in terms of the density of the medium (ρ) and the speed of the sound (*v*):

(1) Z=ρ×v

### 2.2. The Electrodes

From a review of different electrodes for high-temperature sensors [21], platinum was selected and the supplier (INRS) offered to add a layer of titanium to ensure better adhesion of the platinum. The range of thickness for electrodes is around 100 nm, so 100-nm-thick platinum electrodes were deposited with a 20 nm titanium adhesion layer onto the piezoelectric element by physical vapor deposition and were usable up to 900 ${}^{\circ }$C [18]. The type of deposition selected was thermal and electron beam deposition.

### 2.3. The Backing Layer

The backing layer increases the amount of energy radiated to the back of the transducer, reducing the amount of energy in the thickness resonance and increasing the bandwidth, and gives mechanical support to the transducer. The main goal is to extract all the energy coming from the piezoelectric crystal, which does not propagate in the test piece, and to absorb this energy so that it does not reflect back to the crystal. Therefore, the material has to have the same acoustic impedance as the crystal to extract all the energy not transmitted to the part under inspection; see Equation (1). As only part of the crystal is covered by the backing, the resonance of the piezoelectric element is such that the bandwidth is increased but adequate sensitivity is retained. Most backing elements have relatively low acoustic impedance (2.35–15.6 MRayl) [22], which results in an interface. At the interface between two materials, a wave will reflect according to an energy reflection coefficient *R*, and transmit according to an energy transmission coefficient *T*:

(2) $$\begin{array}{cc} R & ={\left(\frac{Z_{p}-Z_{bl}}{Z_{p}+Z_{bl}}\right)}^{2} \end{array}$$

(3) $$\begin{array}{cc} T & =\frac{4\;Z_{p}\;Z_{bl}}{{\left(Z_{p}+Z_{bl}\right)}^{2}} \end{array}$$

where $Z_{p}$ is the acoustic impedance of the piezoelectric material, and $Z_{bl}$ is the acoustic impedance of the backing layer. According to Equation (), to have an energy transmission coefficient *T* with the lithium niobate superior to 85%, the backing layer material must have acoustic impedance between 15.1 and 65.4 MRayl.

Then, this material absorbs all this energy thanks to its acoustic wave attenuation property. The attenuation includes the absorption and the scattering in the material. The acoustic attenuation coefficient defines the material’s attenuation for a given frequency of use; see Equation (4). A technique to quantify this parameter is to compare the amplitude between two successive backwall echoes. The pulse-echo technique, which consists of sending and receiving a wave with the same transducer, allows for obtaining successive backwall echoes.

(4) $$20\;log\;\frac{A^{\prime }}{A^{\prime \prime }}=2\;\hat{\alpha }\;t_{h}$$

where A${}^{\prime }$ is the amplitude of the first echo, A${}^{\prime \prime }$ is the amplitude of the second echo, $\hat{\alpha }$ is the attenuation constant [dB/mm], and $t_{h}$ is the thickness of the medium [mm]. A ratio of 100 between the amplitude of 2 successive echoes allows an acceptable signal-to-noise ratio (SNR). Only one percent of the signal will be reflected. With the idea of having a small transducer, the thickness should not exceed 40 mm. The attenuation coefficient $\hat{\alpha }$ condition must be a minimum of 0.5 dB/mm.

The last criterion to take into account is the temperature variation, i.e., the CTE. Assuming that the piezoelectric crystal and the backing layer are rods, it is possible to calculate that the maximum operating temperature will be 762 ${}^{\circ }$C for steel 304, for example [23]. At room temperature, this backing layer is typically a mixture of epoxy and tungsten. The epoxy allows for strong mechanical resistance to the piezoelectric crystal and attenuates the sound waves. The tungsten content helps to match the acoustic impedance of the piezoelectric crystal and increases the bandwidth of the transducer because this material is dense (ρ= 19.3 g/cm${}^{3}$) [24]; see Equation (1). This mixture also leads to a heterogeneous layer, thus increasing the scattering of the waves thanks to its pores. Epoxy–tungsten mixtures typically have strong attenuation (0.25–5 dB/mm) [22]. This mixture allows for an effective backing layer, affordable and easy to realize, but unfortunately completely unsuitable for high-temperature applications as epoxy cannot be used above 350 ${}^{\circ }$C.

In the present study, adhesive technical ceramics were chosen, as they are able to withstand elevated operating temperatures. The following composition was used: powdered steel 17-4PH, calcium aluminate cement and distilled water are mixed in a proportion of 12:2:1, respectively, and then 0.01 of the weight of superplasticizer is mixed with the paste [25]. The backing was cured in an environmental chamber at room temperature with 100% relative humidity for 28 days. The properties were tested following the procedure of the pulse-echo technique to determine the speed of sound, the acoustic impedance and the acoustic attenuation. The backing layer had a density of 4.43 g/cm${}^{3}$ and a longitudinal speed of sound of 3952 m/s, so its acoustic impedance was 17.5 MRayl, according to Equation (1). Its acoustic impedance was 0.78 dB/mm according to Equation (4). Finally, as stainless steel 17-4PH has a CTE between 10 and 12 × 10${}^{-6}$/${}^{\circ }$C and calcium aluminate cement has a CTE between 8.7 and 13.4 × 10${}^{-6}$/${}^{\circ }$C, the CTE of the mixture was not tested but assumed to be between 8.7 and 13.4, which is close to that of LiNbO${}_{3}$.

### 2.4. The Matching Layer

The matching layer allows for the more efficient transfer of energy from the piezoelectric crystal to the test piece, since the acoustic impedance of the crystal and the test piece is usually different. The main disadvantage is that this layer depends on the wavelength and is therefore optimal for a limited range of frequencies. This reduces the bandwidth of the transducer. It is important to note that as the temperature changes, the acoustic impedance varies due to the change in the ultrasound velocity of the material. Therefore, this acoustic matching should be considered at the temperature of the measurement.

In order to not conflate the results of this study performed at various temperatures, a transducer without a matching layer was used. However, in practice, a matching layer optimized to the particular operating environment may be added to the transducer to improve the efficiency or to avoid bonding the transducer.

### 2.5. Coupling

The emitted waves can be transmitted to the test piece in three ways: dry coupling (with high pressure) [26], liquid coupling (fluid or gel) and solid coupling (foil). The dry coupling is ensured by the sufficient pressure of the transducer on the part to be inspected so that the thickness of the layer of air at the interface is lower than 0.01 mm, according to Drinkwater et al. [27]. This requires the materials to have a high-quality surface finish (<1.63 μm Ra). The liquid coupling is based on water or gel. It is widely used at room temperature and contains rustproof components. However, no couplers are still liquid or viscous after one month at 650 ${}^{\circ }$C. ECHOultrasonics [28] offers a gel that works at 650 ${}^{\circ }$C, but only for a few seconds. Others are oil-based, but burn or are flammable at high temperatures. The present study therefore uses a screwed assembly, allowing for the transducer to be placed on the part to be tested in order to achieve dry coupling. In addition, a silver foil of 50 μm was used to bond the lithium niobate to the test piece.

### 2.6. Other Components and Transducer Assembly

The transducer is composed of a 36${}^{\circ }$ Y-cut lithium niobate piezoelectric crystal with a diameter of 12 mm (such that a resonant frequency is centered at around 3 MHz) and two 100 nm electrodes of platinum with a 20 nm titanium adhesion layer, which was deposited onto the piezoelectric element by physical vapor deposition; a mineral insulated wire composed of two conductors of nickel (Omega) is used to drive the transducer. These wires are bonded to the electrodes and the casing by silver paste (SPI Supplies). The backing layer is a powdered steel 17-4PH with calcium aluminate cement cured directly onto the piezoelectric element. A stainless steel 304 tube is used for the casing, where a cap can be screwed. Table 1 summarizes the selected components of the transducer. The transducer follows Figure 1, except for the matching layer, which is not present, as mentioned previously. A silver foil is used to couple the crystal into the casing with the test piece in an assembly with screws to clamp the transducer to the test piece.

## 3. Experimental Method

Four identical high-temperature transducers (A, B, C and D) were assembled as detailed above and then submitted to different tests until destruction to simulate their use in various industrial environments. The first two tests were designed to demonstrate the maximum operating temperature of the sensor. The furnace temperature was gradually raised until the sensor signal was lost. The third test was for thermal cycling resistance. The last test was a long-term study at high temperatures to show viability in at least a 550 ${}^{\circ }$C environment over several months, with a perturbation at 650 ${}^{\circ }$C for two weeks. This latter has not been demonstrated to date in other scientific papers. The electronic equipment used for the first two tests was high-power equipment to ensure that the sensor worked. For tests three and four, portable equipment was used to demonstrate the viability of the transducer in an industrial or inspector’s environment.

### 3.1. Transducers A and B: Maximum Operating Temperature (MOT) Test

**Figure 2:**
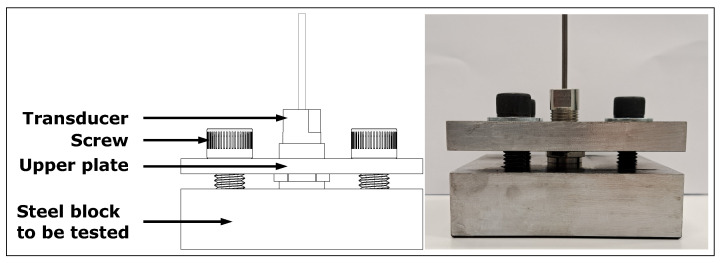
Transducer A or B installed onto low-carbon steel block.

A low-carbon steel block (0.02% wt. C, 1.15% wt. Mn, 0.01% wt. P, 0.01% wt. S, 98.20% wt. Fe) of dimensions 101.6 mm × 101.6 mm × 32.8 mm was inspected to confirm the transducer’s maximum operating temperature. The steel part was polished (1200-grit sandpaper) on the surface in contact with the transducer to ensure good contact. The transducer was clamped using 3 screws, as shown in Figure 2. The tightening of the screws was controlled with a torque wrench and 20 N·m was applied, which is equivalent to a pressure of 49.3 MPa on the transducer.

**Figure 3:**
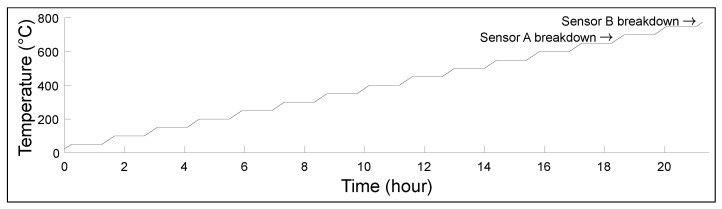
Transducers A and B from room temperature to 750 ${}^{\circ }$C ramp.

This experimental setup was placed in a furnace (Thermolyne 48000) and a pulse-echo method was used to measure the speed of sound of the steel block. The transducer was connected to a signal generator (Agilent 33500B series), which sent a five-cycle Hann windowed toneburst centred at 3 MHz and 1 V. This signal was then amplified to 300 V by an amplifier (Ritec RPR-4000). The signal was received on a receiver (Ritec BR-640), which filtered the signal and amplified it to display it on an oscilloscope (Agilent Technologies DSO9024H). An initial baseline acquisition was taken at room temperature. Clear backwall echoes were observed and used to calculate the speed of sound. As there was no phase transition, twice the thickness of the block was divided by the time difference between the maximum of the first and second backwall. At each temperature, the thickness used was considering the linear thermal dilatation. The furnace was heated to 50 ${}^{\circ }$C, 100 ${}^{\circ }$C, 150 ${}^{\circ }$C, etc., at 2 ${}^{\circ }$C/min. A dwell time of one hour was used prior to measurement at each elevated temperature. The process was repeated for each 50 ${}^{\circ }$C increment until the measurement signal was lost. Figure 3 shows the temperature profile for Transducers A and B, as well as their respective failure temperatures. The break of the crystal caused the transducer breakdown. A sound was heard during the experiment, and the signal was lost simultaneously. Once the experiment was back to room temperature, the assembly was dismantled with precaution, and the backing layer was easily removable as the crystal piezoelement was broken into a thousand pieces. The crystals broke at a temperature far from the Curie temperature due to the coefficient of thermal expansion (CTE). The approach is based on two rods fixed between two massive rigid walls [23]. In our case, if the backing was only stainless steel, the maximal operating temperature before the crystal broke was 762 ${}^{\circ }$C. If the backing layer was only made of calcium alumina cement, the crystal broke at 634 ${}^{\circ }$C. The reality is that the elements are placed in contact together, and then pressure is applied using screws, so we are not far from the hypothesis, but the temperature of the crystal break could be higher.

### 3.2. Transducer C: Thermal Cycling

**Figure 4:**
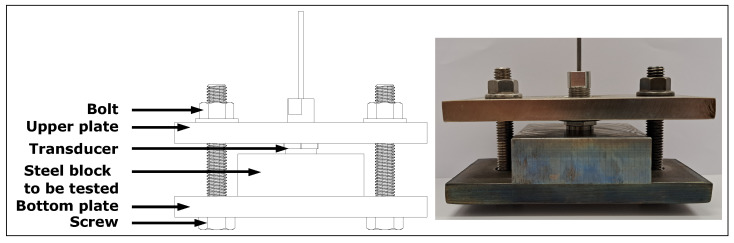
Transducer pressed onto a steel block to be tested on thermal cycles.

A low-carbon steel block (0.24% wt. C, 0.34% wt. Mn, 0.01% wt. P, 0.04% wt. S, 98.90% wt. Fe) of dimensions 101.6 mm × 50.8 mm × 25.4 mm was inspected in order to assess whether or not the transducer could handle thermal cycles. In this case, Tranducer C was coupled to the test piece with a silver foil as a couplant. As before, a screw assembly was used to attach the tranducer to the steel block, as shown in Figure 4.

**Figure 5:**
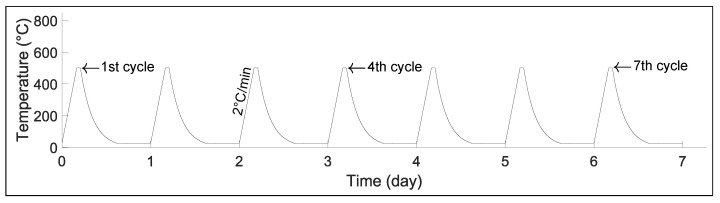
Temperature cycles used to test Transducer C.

Transducer C and the steel block were placed in the same furnace as for the previous experiments. However, in this case, Transducer C was connected to a portable device (Olympus Omniscan SX)—a commercial ultrasonic testing system that acted as a signal generator and analyzer. In this test, the furnace was heated at 2 ${}^{\circ }$C/min from room temperature up to 500 ${}^{\circ }$C. This temperature was maintained for one hour prior to taking the measurement, after which the set-up was allowed to air-cool overnight inside the turned-off furnace. This process was repeated daily for seven days in a row; see Figure 5.

### 3.3. Transducer D: Long Term Performance

**Figure 6:**
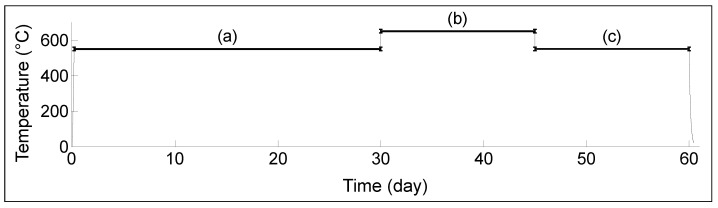
Heating curve for long-term performance: (**a**) one month at 550 ${}^{\circ }$C, (**b**) 14 days at 650 ${}^{\circ }$C, (**c**) 14 days at 550 ${}^{\circ }$C.

The block of low-carbon steel, from the first experiments (0.02% wt. C, 1.15% wt. Mn, 0.01% wt. P, 0.01% wt. S, 98.20% wt. Fe), of dimensions 101.6 mm × 101.6 mm × 32.8 mm, was measured to determine the transducer’s capacity for long-term operation. Transducer D was installed as shown above in Figure 2. Transducer D was also connected to an Omniscan SX portable device. The transducer and steel block were placed inside the furnace and held for one month at 550 ${}^{\circ }$C, followed by 14 days at 650 ${}^{\circ }$C, subsequently followed by another 14 days at 550 ${}^{\circ }$C. This temperature profile is shown in Figure 6.

## 4. Results and Discussion

This section presents the results of the tests performed with the four transducers (A, B, C and D). The first two tests show the maximum operating temperatures of Transducers A and B. The thermal cycling resistance test shows the influence on the signal after seven cycles on Transducer C. The last test shows the viability at 550 ${}^{\circ }$C for one month, followed by 650 ${}^{\circ }$C for two weeks, subsequently followed by another two weeks at 550 ${}^{\circ }$C on Transducer D.

### 4.1. High-Temperature Performance

**Figure 7:**
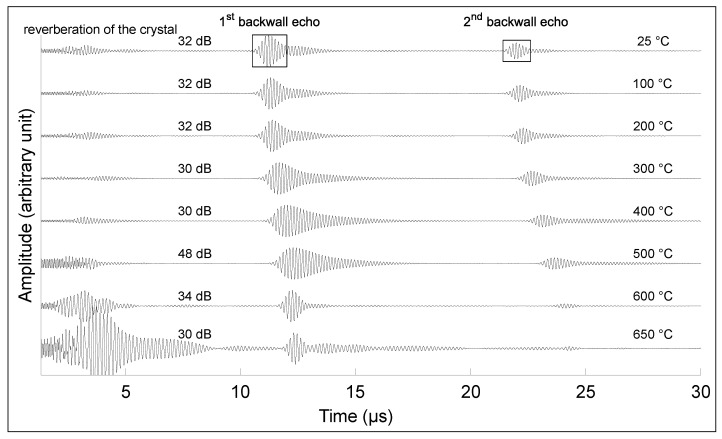
Signal amplitude on 32.8-mm-thick low-carbon steel block for temperatures between 25 ${}^{\circ }$C and 650 ${}^{\circ }$C, showing the first and second backwall echoes from Transducer A.

**Figure 8:**
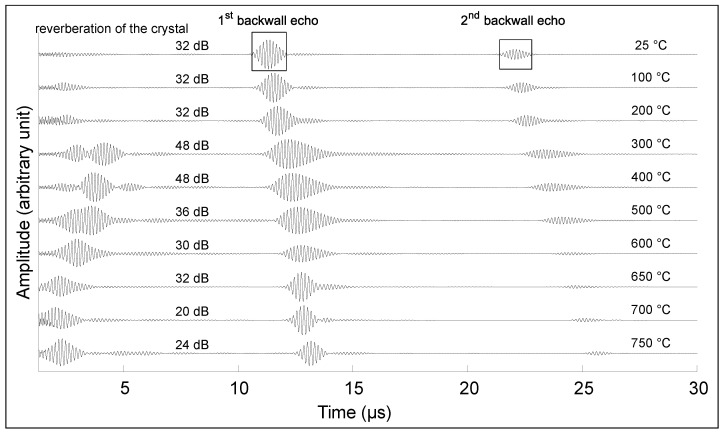
Signal amplitude on 32.8-mm-thick low-carbon steel block for temperatures between 25 ${}^{\circ }$C and 750 ${}^{\circ }$C, showing the first and second backwall echoes from Transducer B.

Figure 7 and Figure 8 show the first and second echoes of the test piece from room temperature to the temperature breakdown for Transducer A and Transducer B, respectively, at 650 ${}^{\circ }$C and 750 ${}^{\circ }$C. The amplitude of each signal was normalized on the first echo for each temperature, and the gain of the receiving amplifier in dB is provided. Two phenomena can be observed: the amplitude varies nonlinearly with the temperature, as does the bandwidth. In the case of Transducer B, no change in the signal was observed after 72 h at 550 ${}^{\circ }$C. However, upon further heating at 2 ${}^{\circ }$C /min, the crystal broke once 785 ${}^{\circ }$C was reached, and no signal was observable anymore.

The time of the backwall echo shifts with temperature indicates a reduction in the longitudinal wave velocity, mainly due to changes in the elastic constants. The velocity reduces from 5904 m/s at 25 ${}^{\circ }$C to 5320 m/s at 650 ${}^{\circ }$C. This reduction is consistent with the literature’s values for low-carbon steel [34,35,36]. A thermocouple must be added to correct the value of the velocity in order to avoid inaccuracy in the thickness value.

### 4.2. Thermal Cycling

**Figure 9:**
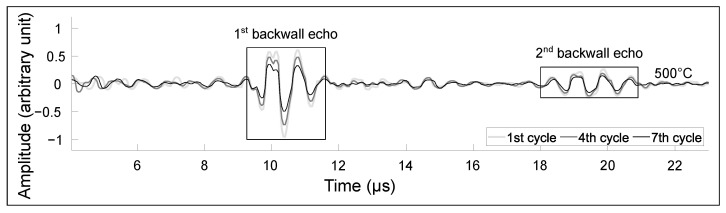
Signals obtained at three different stages of the thermal cycling experiment. The signals shown were obtained after one hour at 550 ${}^{\circ }$C. The thick light grey line was obtained during the first cycle, the dark grey lines during the fourth cycle and the thin black line during the seventh cycle.

Figure 9 shows the first and second echoes of the test piece for representative cycles. Each signal is normalized on the maximum of the absolute value of the first echo of the first cycle for easier comparison. The signal is truncated to highlight the first and second echoes. With the number of cycles, the maximum amplitude of the signal reduces by 2.3 dB between the first and the fourth cycle and 5.7 dB between the first and seventh cycles; from an operational standpoint, this can be compensated by increasing the gain of the amplifier. The loss in amplitude could be due to the creep and stress relaxation in the steel assembly with thermal cycling, as the screws were not re-tightened between cycles.

### 4.3. Long-Term Performance

**Figure 10:**
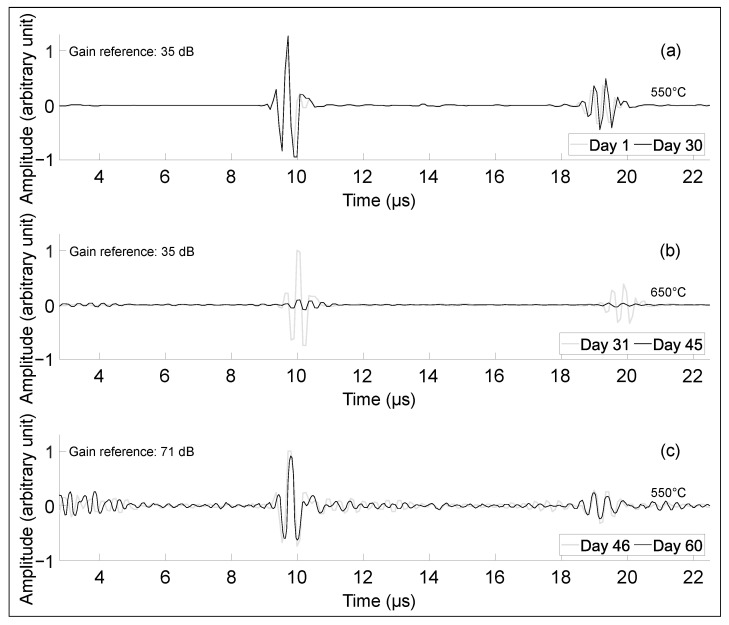
Transducer performance is maintained during long-term high-temperature exposure: (**a**) echoes on day 30 taken at 550 ${}^{\circ }$C (550 ${}^{\circ }$C for 30 days), (**b**) echoes on day 44 taken at 650 ${}^{\circ }$C (550 ${}^{\circ }$C for 30 days followed by 650 ${}^{\circ }$C for 14 days) and (**c**) echoes on day 68 taken at 550 ${}^{\circ }$C (550 ${}^{\circ }$C for 30 days followed by 650 ${}^{\circ }$C for 14 days followed by 550 ${}^{\circ }$C for 14 days).

Figure 10 shows the performance of the transducer after being held at 550 ${}^{\circ }$C for one month; no signal degradation was observed. After exposure to 650 ${}^{\circ }$C for two weeks, the signal decreased by 36 dB. This could be explained by the fact that the carbon steel oxidized at 650 ${}^{\circ }$C; this could also be due to lower pressure on the transducer—with the thermal dilatation, the pressure is reduced so the amplitude drops. Disc springs could be used to compensate for thermal expansion during the test [26]. Upon cooling back down to 550 ${}^{\circ }$C, the transducer did not show further degradation. No significant loss in performance was observed, indicating the potential application of this high-temperature piezoelectric design for continuous inspection at elevated temperatures.

Stainless steel alloys should be preferred at high temperatures to sustain such high temperatures. In this study, ferritic alloys were used instead of austenitic alloys. Ferritic stainless steels have a body-centered cubic (BCC) crystal structure and are magnetic, while austenitic stainless steels have a face-centered cubic (FCC) crystal structure and are non-magnetic. Ferritic stainless steel tends to have lower attenuation coefficients for ultrasound than austenitic stainless steel, making it easier for the ultrasound to travel through. This is because ferritic stainless steel has a lower density and elastic modulus than austenitic stainless steel. Therefore, ferritic stainless steel is more transparent to ultrasound than austenitic stainless steel. This sensor should then be used on an austenitic steel alloy to validate its performance over a longer period of time.

## 5. Conclusions

An ultrasound transducer design composed of a lithium niobate piezoelectric element, two Pt/Ti electrodes, a mineral insulated wire, a metal-ceramic composite backing layer and no matching layer was bonded by a silver foil to a low-carbon steel block using a screw clamp assembly. We found that such a transducer design was able to successfully monitor the steel block thickness, in situ, at high temperatures. This performance was maintained even after long-term (2 months) high-temperature exposure. The piezoelectric transducer without an acoustic coupling layer used was capable of measuring the thickness of a 32 mm steel block in a 750 ${}^{\circ }$C environment for at least 72 h, able to operate several thermal cycles and survive in an environment of at least 550 ${}^{\circ }$C for a couple of months. These results confirm the viability of a piezoelectric ultrasound transducer that can operate for extended periods at temperatures significantly higher than had previously been reported.

## Figures and Tables

**Table 1:** Composition and properties of the selected materials.

Layer	Composition	Dimension	Acoustic Impedance [MRayl]	Specific Characteristics	MOT [^∘^C]	CTE [10${}^{-6}$/${}^{\circ }$C]	Ref.
Wires	Mineral insulated cable with Ni wire	Diameter 1.57 mm Length 15 cm	50	N/A	650	12.8	[29]
Junction	Silver paste	A few drops	37.8	N/A	962	18.8	[30]
Electrodes	Pt/Ti	100 nm/20 nm	N/A	N/A	650	N/A	[21]
Piezoelement	LiNbO${}_{3}$ 36${}^{\circ }$ Y-cut	Diameter 12 mm Thickness 1.22 mm	34	k${}_{22^{\prime }}$ = 48.5% d${}_{22^{\prime }}$ = 38.5 pC/N	1210	15.4 ${}^{1}$ 7.5 ${}^{2}$	[19]
Backing Layer	Powdered steel 17-4PH Calcium aluminate cement Distilled water	Diameter 15 mm Thickness 30 mm	17.6	Acoustic attenuation 0.54 dB/mm	850	8.7–13.4	[31]
Casing	Stainless steel 304	Diameter 15 mm Length 35 mm	45	0.06 dB/mm	925	17.2	[32]
Matching Layer ${}^{3}$	Steel 400 series	Thickness $\frac{\lambda }{4}$	45	0.05 dB/mm	705	10–12	[33]

^1^ Coefficient of thermal expansion parallel to C-axis. ^2^ Coefficient of thermal expansion perpendicular to C-axis. ^3^ Not considered for this study.

## Data Availability

The experimental data obtained in this study are available from the authors by reasonable request.
